# Prevalence of tobacco dependence and associated factors among patients with schizophrenia attending their treatments at southwest Ethiopia; hospital-based cross-sectional study

**DOI:** 10.1371/journal.pone.0261154

**Published:** 2021-12-15

**Authors:** Defaru Desalegn, Zakir Abdu, Mohammedamin Hajure

**Affiliations:** Department of Psychiatry, College of Health Sciences, Mettu University, Mettu, Oromia Region, Ethiopia; Chiba Daigaku, JAPAN

## Abstract

**Background:**

Tobacco smoking is the most typically employed in patients with mental disorders; among them, patients with schizophrenia are the very best users. The rate of smoking among patients with schizophrenia is between two and three times greater than the general population in western countries. However, there is a scarcity of studies on the magnitude and associated factors of tobacco dependence among patients with schizophrenia in Ethiopia. Therefore, we assessed the prevalence of tobacco dependence and associated factors among patients with schizophrenia at Mettu Karl referral, Bedelle, and Agaro hospitals, Southwest, Ethiopia.

**Method:**

Hospital-based the multistage stratified cross-sectional study design was conducted among 524 patients with schizophrenia who are on treatment. Fagerstrom Test for Nicotine Dependence (FTND) was used to screen the prevalence of tobacco dependence. Analysis of data was done using SPSS version 24.

**Result:**

The prevalence of tobacco dependence among study participants was 22.3% (95% CI) (18.6, 26). Concerning the severity of tobacco dependence, 3.5%, 13.8%, and 5% of the respondents report moderate, high, and very high levels of tobacco dependence respectively. The proportions of tobacco dependence among male schizophrenic patients 88 (25.8%) were higher compared to their counterparts 27 (15.5%). After controlling the effects of cofounders in the final regression analysis, male gender (AOR 2.19, 95% CI = 1.25, 3.83), being on treatment for more than 5years (AOR 4.37, 95% CI = 2.11, 9.02), having a history of admission (AOR 4.01, 95% CI = 1.99, 8.11), and family history of mental illness (AOR 1.90, 95% CI = 1.04, 3.48) were shown to have a significant positive association with tobacco dependence.

**Conclusion and recommendation:**

A study show a significant proportion of tobacco dependence among people living with schizophrenia. Factors like, being male gender, being on treatment for more than 5 years, having a history of admission, and family history of mental illness was found to have a significant positive association with tobacco dependence. Hence, there is a need for coordinated and comprehensive management clinically to manage tobacco dependence along with identified risk factors in patients with schizophrenia. Also the finding call for the clinicians, managers, ministry of health and other stakeholders on the substance use prevention strategies that target personal and environmental control.

## Introduction

Globally, cigarette smoking is among the highest 5 causes of risk mortality and is that the single largest preventable reason behind death; it promotes quite five million annual deaths, inflicting 11% of ischemic heart deaths and quite seventieth of respiratory organ, cartilaginous tube, and trachea cancer [1]. According to the report of WHO, cigarette smoking in the developed countries is the cause of 20% of preventable death [2]. The report shows that tobacco is estimated to kill about one billion people in the 21^st^ century, particularly from low- and middle-income countries (LMICs) [3].

People living with mental illness are more likely to smoke and be at greater risk for smoking‐related health problems than the general population [4]. Mortality from smoking is higher among individuals living with mental illness supported by the report of the study comprised 600,000 respondents where tobacco-related conditions were contributed to 53% of total deaths in schizophrenia [5]. Smoking in schizophrenic patients contributes to a 20% decrease in their life expectancy compared to members of the general population [6].

The impact of smoking among patients with schizophrenia not only increases metabolism and vascular risks [7], also increases suicide risk [8]. It decreases the antipsychotic therapeutic effects as smoking induce the medication metabolism in the liver reducing up to 48% of the active metabolites in serum [9]. Schizophrenic-smokers show more hospitalization frequency (than schizophrenic non-smokers) and also require more depot medication, having fewer adherences to treatment [10].

A meta-analysis of 42 epidemiological studies across 20 different countries showed that people with schizophrenia have more than five times the odds of current smoking than the general population and smoking cessation rates are much lower in smokers with schizophrenia compared with the general population [11]. Another meta-analysis study was done in 8 countries based on 14 studies found that the average prevalence of current smokers among male schizophrenia patients were 72% [12].

According to one study from the United Kingdom (UK) done among 8 million patients, the prevalence of smoking among psychotic patients (schizophrenia, schizotypal and delusional disorders) was 44.6% [13].

A cross-sectional study was done in China among inpatient schizophrenic patients found that the prevalence rate of current smoking was 40.6%, which was 57.5% in males and 6.3% in females and the study described that factors such as being male sex, older age, poor marital status, alcohol use, use of first-generation antipsychotics, longer duration of illness, more frequent hospitalizations, and more severe negative symptoms were independently associated with current smoking [14].

A cross-sectional study was done in Singapore among male schizophrenic patients found that the lifetime prevalence of smoking cigarettes and current smokers are 54.1% and 42.4% respectively [15], in Iran as high as 71.6% [16], in Scotland 53.4% [17], and in Turkey 49% [18].

One a cross-sectional descriptive study was done in Southwest Nigeria among 367 patients with schizophrenia found that the lifetime prevalence and a current smoking rate of 20.4% and 25.9% were reported respectively [19]. Facility-based a cross-sectional study done in Jimma medical center in Ethiopia on tobacco dependence among people with mental illness found that the prevalence of current tobacco dependence among the study participants is 18.5% and specifically the prevalence of tobacco dependence among patients with schizophrenia was 29.1%; furthermore, the study described their level of tobacco dependence as 57.7% moderate, 29.5% higher and 12.8% very high [20].

Little information is available regarding nicotine dependence among patients with schizophrenia in Ethiopia. Thus, this study was aimed to assess the prevalence of tobacco dependence and associated factors among patients with schizophrenia attending their treatments at Mettu Karl, Bedelle, and Agaro hospitals, Southwest Ethiopia.

## Materials and methods

### Study setting and period

The study was conducted from 1^st^ April to 30^th^ June 2019 at the psychiatric clinic of three governmental health institutions (Hospitals) found in southwest Ethiopia, namely Mettu Karl referral, Bedelle and Agaro hospitals, which were 600 kilometer, 426 kilometer, and 397 kilometer far from Addis Ababa to the southwest, the capital city of Ethiopia, respectively.

### Study design

Hospital-based a cross-sectional study was conducted.

### Source population

All patients with schizophrenia attending follow-up treatments at Mettu Karl referral, Bedelle, and Agaro hospitals psychiatric clinic

### Study population

Sample of patients with schizophrenia who attended the outpatient treatment at the psychiatric clinic of Mettu Karl referral, Bedelle, and Agaro hospitals during the data collection period

#### Inclusion and exclusion criteria

Adult patients (aged 18 and above) with schizophrenia who were already diagnosed previously as per the diagnostic criteria of the Diagnostic Statistical Manual of Mental Disorders, 4^th^ and or 5^th^ edition (DSM-IV or DSM-V) were included in the study and patients with schizophrenia whose illness was in the acute stage or in exacerbation of symptoms were excluded from the study.

### Sample size determination

The minimum the sample size required for this study was determined by using the formula to estimate single population proportion, n = ((z ᾳ/2)^2^p(1-p))/d^2^ by using the following assumptions: the prevalence of tobacco dependence among patients with schizophrenia at the Jimma medical center was 29.1% [20], a 95% confidence interval (CI), 5% the margin of error and a non-response rate of 10%. We applied the single population proportion formula to give in = (1.96)^2^ * 0.291 (1–0.291) / (0.05)^2^ = 317.

Since multistage stratified sampling the technique was used to select study participants, using design effect the sample size was multiplied by 1.5, giving 476 considering that the questionnaire was self-administered and finally adding a 10% non-response rate, the final number of the study subject became 524.

### Sampling technique

**Fig 1:**
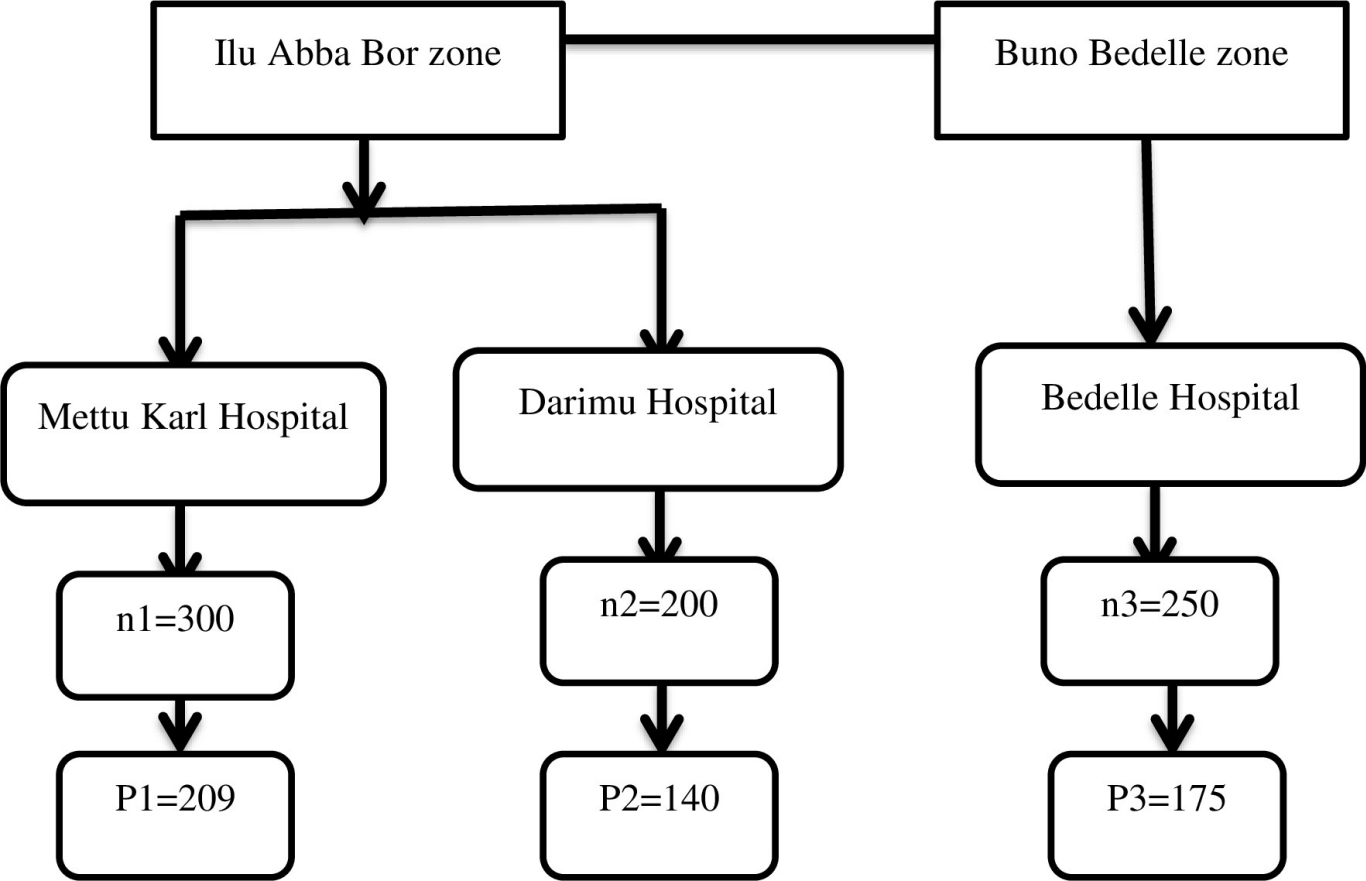
The schematic presentation of the sampling procedure that was employed to select study participants from three zones, southwest, Ethiopia, 2019. Where, n–is the average number of schizophrenic patients who were treated at the psychiatric clinic of each hospital per one month (data collection period) as reviewed from the patients’ registration book. P–is the number of schizophrenic patients who are allocated proportionally to the hospitals.

The multi-stage stratified sampling technique was used to select the study participants. Stratification was first done on the zone level, then by the hospitals found in the zones (**Fig 1**).

### Data collection procedure and tools

An interviewer-administered a structured questionnaire was used to collect information. Questionnaires about demographic and other clinical factors were developed after an extensive review of the literature and similar study tools. We employed nine interviewers’ (data collectors) for 2 months data collection period for collecting data from the participants (patients with schizophrenia). The interviewers’ background or expertise was that they were all bachelors of degree and master of degree holders in psychiatric nursing. Hence, we (authors) believe that the interviewers’ background or expertise (being psychiatric professionals) can lead them to determine the capacity of patients to provide consent for the study. The study was done among patients with schizophrenia who were already diagnosed previously as per the diagnostic criteria of the Diagnostic Statistical Manual of Mental Disorders, 4^th^ and or 5^th^ edition (DSM-IV/DSM-V) and currently attending their treatments at health facilities (hospitals). The diagnosis of the patients was first confirmed by reviewing patients’ cards prior to starting data collection (interviewing the patients) and also interviewer’s perception of the patient capacity was determined based on the patient level of remission. Fagerstrom Test for Nicotine Dependence (FTND) has six items, with a total score ranging from 0 to 10 was used to measure nicotine dependence [21]. The FTND has been shown to have good test-retest reliability and validity in populations of smokers with mental health problems [22]. At a cut-off score ≥of 5, the FTND has good sensitivity and specificity (0.75 and 0.80, respectively) [23] and was considered to indicate tobacco dependence.

Alcohol use disorders (AUDs) were assessed using the four-item CAGE questionnaire (Cut down, Annoyed, Guilty, and Eye-opener). CAGE is short and easily applied in clinical practice. The sensitivity and specificity of CAGE at a cut-off score ≥ 2 was 0.71 and 0.90, respectively [11]. In this study, a total score ≥2 on CAGE was used to indicate an alcohol use disorder.

The severity of Dependence Scale (SDS) was used to assess Khat use disorder. It is a screening tool for the Diagnostic and Statistical Manual of Mental Disorders, Fifth Edition (DSM-5) ‐ defined Khat use disorder [24]. SDS is a brief and simple screening tool that was validated in Mizan, the Southwestern part of Ethiopia to identify individuals experiencing a Khat use disorder syndrome and experiencing high rates of adverse consequences in association with the use [25]. Each of the five items is scored on a 4-point scale (0–3). The total score is obtained through the addition of the 5-item ratings.

### Data processing and analysis

Epi Data Version 3.1 was used for data entry following checks and coded for. Then, the data were exported to the Statistical Package for Social Science Version 24.0 for further analysis. Simple descriptive statistics (median, percentage, frequencies, and interquartile range) were used to compute demographic characteristics of participants. In addition, bivariable analysis was used to see the significance of the association. Variables that showed strong association (p-value <0.25) in bivariate analysis were entered into multivariable logistic regressions to identify independently associated variables. Multicollinearity was checked by the Variance Inflation Factor (VIF). Statistical significance was declared at a p-value less than 0.05. The significance of the association of the variables was described using Adjusted Odds Ratio (AOR) with a 95% confidence interval.

### Data quality control

The questionnaire was prepared first in English and translated into Afaan Oromo/Amharic then back-translated to English by another person who was blinded for the English version to check the clarity of the questionnaire. To identify potential problems and to make important modifications, the questionnaire was pre-tested on 5% of the total study participants were randomly selected in the same population outside the study area in Jimma Medical center psychiatric clinic one week before the actual data collection date. The prepared questionnaire was checked thoroughly for its completeness, objective, and variable before it was distributed to respondents. Also, the collected data were checked for its completeness. The supervisor was three first-degree holder instructors. A pre-test was done after training is given to the supervisors on how to supervise data collection. The principal investigator checked for the completeness of filling questionnaires at the end of each data collection date. Any error, ambiguity, incompleteness, or another encountered problem was addressed immediately after the supervisor receives the filled questionnaire from each data collector.

### Operational definition

**Tobacco dependence =** individuals who score FTND 5 and above.

- A total FTND score of five indicates moderate nicotine dependence,
- A a score of 6–7 indicates high nicotine dependence and
- A a score of 8–10 indicates very high nicotine dependence

**Schizophrenia:** is a clinical diagnosis reached by a clinician based on DSM-IV/DSM-5 diagnostic criteria as reviewed from the patient card.

**Physical illness:** is any diagnosed medical problem like hypertension, diabetes Mellitus, heart failure made by the clinician during the follow-up period.

**Substance use:** ever use of any psychoactive substance in the past 12 months.

### Ethical clearance

Ethical clearance was obtained from the Research, Ethical Review Board of Mettu University, college of health sciences, and the study was done according to the declaration of Helsinki. And also an approval letter was obtained from the head department of psychiatry. After the ethical review board has approved the consent procedure, selected participants were told about the nature, purposes, benefits, and adverse effects of the study and invited to participate. Participants were told the right to refuse or discontinue participation at any time they want. Confidentiality was ensured and all related questions, they raised were answered during data collection. Written informed consent was obtained from study participants.

## Results

### Socio-demographic characteristics of the study participants

A total of 524 participants was participating in the study, of which 515 responded, giving a response rate of 98.3%. The mean age (±SD) of the study, participants were 33.7 (±7.9) years of age. About 287 (55.7%) of the respondents were married. Among the respondents, 341 (66.2%) were male, 326 (63.3%) were Oromo, and 150 (29.1%) of the study participants had attended primarily (grade 1–8) education More than half of them had a family size of four or above and the median monthly incomes of the respondents were 700ETB, which ranges from 100-5000ETB. (**Table 1**)

**Table 1:** Socio-demographic characteristic of patients with schizophrenias at southwest Ethiopia, 2019 (n = 515).

Variables	Category	Numbers (n)	Percentage (%)
Sex	Male	341	66.2
	Female	174	33.8
Age(in years)	18–24	59	11.5
	25–34	215	41.7
	35–44	178	34.6
	45–54	63	12.2
Religion	Muslim	332	64.5
	Orthodox	101	19.6
	Protestant	82	15.9
Ethnicity	Oromo	326	63.3
	Amhara	116	22.5
	SNNP	73	14.2
Marital status	Single	175	34.0
	Married	287	55.7
	Divorced/widowed	53	10.3
Educational status	No formal education	132	25.6
	Primarily school	150	29.1
	Secondary	116	22.5
	Above secondary	117	22.7
Residence	Urban	292	56.7
	Rural	223	43.3
Occupation	Government employed	103	20.0
	Self-employed	185	35.9
	Unemployed	227	44.1
Family size	<4	211	41.0
	≥4	304	59.0
Monthly Income	<700 ǁ ETB	264	51.3
	≥700 ETB	251	48.7

SNNP (South nations and nationalities and peoples)–stands for Kaffa, Dawuro, Yem, Walayta, Gurage and Silte, Median of monthly income, ETB–Ethiopian birr.

#### Clinical and other substance-related characteristics of patients with schizophrenia

More than half of the participants were attending their treatment for less than 6years (56.7%). Of patients with a history of admission, about 17.1% were admitted 2times for their condition. Few of them had both family history of mental illness (15.7%) and substance use (17.1%). About one third (36.3%) of the study participants fulfilled alcohol use disorder using CAGE criteria. (**Table 2**)

**Table 2:** Clinical and other substance-related characteristics of patients with schizophrenia, southwest Ethiopia, 2019 (n = 515).

Variables	Category	Numbers (n)	Percentage (%)
Admission history	No	308	59.8
	Yes	207	40.2
Frequency of admission	1	75	14.6
	2	88	17.1
	3	44	8.5
Physical illness	Yes	38	7.4
	No	477	92.6
Family history of mental illness	No	434	84.3
	Yes	81	15.7
Family history of substance use	No	427	82.9
	Yes	88	17.1
Alcohol use disorder	Yes	187	36.3
	No	328	63.7
Khat dependence	Yes	155	30.1
	No	360	69.9
Duration of illness	<6 years	292	56.7
	≥6 years	223	43.3
Treatment duration	<5 years	271	52.6
	≥5 year s	244	47.7

### Prevalence of tobacco dependence among patients with schizophrenia

The prevalence of tobacco dependence among patients with schizophrenia was 22.3% 95% CI (18.6, 26). Concerning the severity of tobacco dependence, about 3.5%, 13.8% and 5% of the respondents use moderate, high, and very excessive levels of tobacco dependence respectively. (**Table 3**)

**Table 3:** Level of tobacco dependence and frequency of smoking amongst patients with schizophrenia attending their treatments at southwest Ethiopia, 2019 (n = 515).

Variables		Numbers (n)	Percentage (%)
Level of dependence	Moderate (5)	18	3.5
	High (6–7)	71	13.8
	Very high (8–10)	26	5
Frequency of smoking	Never	401	77.9
	Once or twice	34	6.6
	Daily or almost daily	37	7.2
	Weekly	21	4.1
	Monthly	22	4.3

More than half of the respondents, 308 (59.8), smoked less than 10 items of cigarettes on a daily basis. About one-fourth of schizophrenic patients with tobacco dependence smoke cigarettes within 5-30miutes soon after waking. The proportions of tobacco dependence among male schizophrenic patients 88 (25.8%) were higher compared to their counterparts 27 (15.5%).

### Correlates of tobacco dependence among patients with schizophrenia

In the univariable logistic regression, different factors have been shown to have associated with tobacco dependence among patients with schizophrenia. Accordingly, male gender, unemployment, being on treatment for 5years, having a history of admission and frequent admission, presence of physical illness, family history of mental illness, being educated above secondary school

After controlling for cofounders, male gender (AOR 2.19, 95% CI = 1.25, 3.83), being on treatment for more than 5years (AOR 4.37, 95% CI = 2.11, 9.02), having a history of admission (AOR 4.01, 95% CI = 1.99, 8.11), and family history of mental illness (AOR 1.90, 95% CI = 1.04, 3.48) were shown to have a significant positive association with tobacco dependence in the final regression analysis. (**Table 4**)

**Table 4:** Factors associated with tobacco dependence among schizophrenic patients at Mettu Karl referral, Bedelle and Agaro hospitals, southwest Ethiopia, 2020 (N = 515).

Variable	Category	Tobacco use		COR, 95% (CI)	AOR, 95% (CI)
		Not dependent N (%)	Dependent N (%)		
Sex	Female	147 (84.5)	27 (15.5)	Ref	Ref
	Male	253 (74.2)	88 (25.8)	1.89 (1.18,3.05)	2.19 (1.25,3.83)**
Occupational status	Government employed	79 (76.9)	24 (23.3)	Ref	Ref
	Self-employed	168 (90.8)	17 (9.2)	0.33 (0.17,0.66)	0.41 (0.17, 1.00)
	Unemployed	153 (67.4)	74 (32.6)	1.59 (0.93,2.72)	2.09 (0.98, 4.49)
Frequency of admission	None	146 (71.9)	57 (28.1)	Ref	Ref
	1	124 (80.0)	31 (20.0)	0.79 (0.43,1.45)	1.32 (0.66, 2.65)
	2	92 (81.4)	21 (18.6)	0.55 (0.29,1.03)	1.63 (0.66,4.01)
	3	38 (86.4)	6 (13.6)	0.46 (0.19,1.12)	0.67 (0.23,2.15)
Duration of treatment	<5years	220 (81.2)	51 (18.8)	Ref	Ref
	≥5years	180 (73.8)	64 (26.2)	1.53 (1.01,2.33)	4.37 (2.11,9.02)***
Educational status	No formal education	112 (84.8)	20 (15.2)	Ref	Ref
	Primarily school	117 (78.0)	33 (22.0)	1.58 (0.86, 2.92)	0.59 (0.25, 1.44)
	Secondary	88 (75.9)	28 (24.1)	1.78 (0.94,3.37)	0.91 (0.41,2.00)
	Above	83 (70.9)	34 (29.1)	2.29 (1.23,4.27)	0.73 (0.34,1.59)
Admission history	No	265 (86.0)	43 (14.0)	Ref	Ref
	Yes	135 (65.2)	72 (34.8)	3.29 (2.14,5.06)	4.01 (1.99, 8.11)**
Physical illness	Yes	25 (65.8)	13 (34.2)	1.91 (0.95,3.87)	1.79 (0.73,4.43)
	No	375 (78.6)	102 (21.4)	Ref	Ref
Alcohol use disorder	Dependent	151 (80.7)	36 (19.3)	1.33 (0.86,2.07)	1.12 (0.67,1.85)
	Non-dependent	249 (75.9)	79 (24.1)	Ref	Ref
Family history of mental illness	No	347 (80.0)	87 (20.0)	Ref	Ref
	Yes	53 (65.4)	28 (34.6)	2.11 (1.26,3.53)	**1.90** (1.04,3.48)*

Abbreviations: OR, Odds Ratio; CI, Confidence Interval. Ref: Reference category NB.*Persisted significant at P-value <0.05, ** significant at P-value ≤0.001. *** Significant at P-value <0.0001.

## Discussion

A cross-sectional study was conducted in three hospitals located in the southwestern part of Ethiopia revealed about one-quarter of patients with schizophrenia reported tobacco dependence. As the majority of the previously conducted studies targeted cigarette smoking among patients with schizophrenia, however, the current study aimed to determine the prevalence of tobacco dependence and its correlates among patients with schizophrenia. So, this could have additional benefits set direction or develop strategies to deal with the impacts of the problem.

The overall prevalence of tobacco dependence among schizophrenic patients was 25.9%. These results were higher compared to the prevalence of tobacco dependence among the general population in Ethiopia which is 7.9% [26]. This difference could be due to the chronic nature of the illness and is used as a form of self-medication, normalizing some central nervous system deficits involved in the disorder. The results were also higher compared to the finding of the study from Nigeria (20.4%) [19]. The possible difference might be explained due to differences in study instruments (FTND vs. PSE-10). However, the finding of the current study was lower than the result of a study from Turkey 49% [18], United Kingdom 44.6% [13], India 68.8% [27] and Ethiopia 29.1% [20]. The discrepancy might be attributable to the difference in study design, instruments, and study setting.

After controlling for confounders, the odds of developing tobacco dependence among patients who attended treatment for more than 5 years were 4.4 times higher than those who attended for less than 5years. This was in agreement with the findings of the study conducted in China [28] and the USA [29]. It is clear that, from the nature of Schizophrenia, at the time of treatment progress or illness become episodic, the intensity increase over time, exposing them to use tobacco in the form of self-medication [30]. Generally, the longer the duration of the treatment period, the higher the danger of developing tobacco dependence.

Individual patients having a family history of mental illness were 1.9times more likely to develop tobacco dependence compared to those without a family history of mental illness which was supported by the finding of previously conducted studies [31]. The notion was indicating the role of genetic factors in the etiology of smoking behaviour and the high comorbidity between nicotine dependence and schizophrenia [32].

Furthermore, gender was shown a significant association with tobacco dependence in patients living with schizophrenia was gender. Accordingly, in the current study, males were 2times more likely to develop tobacco dependence compared to their counterparts and this was in agreement with the finding of the previous study [14, 33]. The possible reason might be related to the fact that male schizophrenic patients were more likely to smoke tobacco as they experience a lesser intensity of negative symptoms compared to females [34].

Finally, the current study revealed patients with schizophrenia who was a history of admission were more likely to develop tobacco dependence compared to those attending their treatment on an out-patient basis. This was in line with the findings of the previously published studies [14]. The finding of the study supports an association of illness severity with admission history [35].

### Limitations

One of the limitations of this study might be the cross-sectional study design, which does not allow causal inference. Again In the current study, patients who were living with schizophrenia and schizophrenia-like disorder attending inpatient care were excluded. Thus, the finding of the study may not be generalizable to all patients with schizophrenia or like disorder. Also, the lack of any scale measuring current psychopathology was another limitation of this study.

## Conclusions

A study shows a significant the proportion of tobacco dependence among people living with schizophrenia. Factors like, being male gender, being on treatment for more than 5 years, having a history of admission, and family history of mental illness was found to have a significant positive association with tobacco dependence. Hence, there is a need for coordinated and comprehensive management clinically to manage tobacco dependence along with identified risk factors in patients with schizophrenia. Also the finding call for the clinicians, managers, ministry of health and other stakeholders on the substance use prevention strategies that target personal and environmental control.

## Abbreviations

AUD: Alcohol Use Disorder
CAGE: Cut down, Annoyed, Guilty, an Eyeopener
CI: Confidence Interval
FTND: Fagerstrom Test for Nicotine Dependence
LMICs: Low- and Middle-Income Countries
OR: Odds Ratio
SPSS: Statistical Package for Social Science
UK: United Kingdom
WHO: World Health Organization

## References

[1] World Health Organization. WHO report on the global tobacco epidemic, 2009: implementing smoke-free environments. Geneva: World Health Organization; 2009.

[2] World Health Organization, WHO Tobacco Free Initiative. World No Tobacco Day 2008: tobacco-free youth.

[3] Jamison DT, Jha PK, Sankaranarayanan R, Horton SE. Disease control priorities: -cancer. The World Bank; 2015 Jan 1. doi: 10.1038/nrc4019 26913318

[4] Goff DC, Cather C, Evins AE, Henderson DC, Freudenreich O, Copeland PM. Medical morbidity and mortality in schizophrenia: guidelines for psychiatrists. Journal of Clinical Psychiatry. 2005 Feb 15;66(2):183–94. doi: 10.4088/jcp.v66n0205 15705003

[5] Collins A, Ajayi O, Diamond S, Diamond W, Holroyd S. Tobacco Use and Associated Factors in Patients Presenting to a Psychiatric Emergency Room. Journal of addiction. 2018 Jun 13;2018. doi: 10.1155/2018/8102165 30009080 PMC6020520

[6] Colton CW, Manderscheid RW. Congruencies in Increased Mortality Rates, Years of Potential Life Lost, and Causes of Death Among Public Mental Health Clients in Eight States. Prev chronic Dis. 2006;3(2):1–14. PMC1563985 16539783

[7] Hagman BT, Delnevo CD, Hrywna M, Williams JM. Tobacco use among those with serious psychological distress: results from the national survey of drug use and health, 2002. Addictive behaviors. 2008 Apr 1;33(4):582–92. doi: 10.1016/j.addbeh.2007.11.007 18158218 PMC2696205

[8] Malone KM, Waternaux C, Haas GL, Cooper TB, Li S, Mann JJ. Cigarette smoking, suicidal behavior, and serotonin function in major psychiatric disorders. Am J Psychiatry. 2003 Apr;160(4):773–9. doi: 10.1176/appi.ajp.160.4.773 12668368

[9] Mayerova M, Ustohal L, Jarkovsky J, Pivnicka J, Kasparek T, Ceskova E. Influence of dose, gender, and cigarette smoking on clozapine plasma concentrations. Neuropsychiatr Dis Treat. 2018;14:1535–43. doi: 10.2147/NDT.S163839 29950838 PMC6011879

[10] Šagud M, Mihaljević-Peleš A, Mück-Šeler D, Pivac N, Vuksan-Ćusa B, Brataljenović T, et al. Smoking and schizophrenia. Psychiatria Danubina. 2009 Jun 25;21(3):371–5. 19794359

[11] Lindblom EN. Effectively regulating e-cigarettes and their advertising-and the first amendment. Food & Drug LJ. 2015;70:55. 26292472

[12] De Leon J, Diaz FJ. A meta-analysis of worldwide studies demonstrates an association between schizophrenia and tobacco smoking behaviors. Schizophrenia research. 2005 Jul 15;76(2–3):135–57. doi: 10.1016/j.schres.2005.02.010 15949648

[13] Rojas G, Gaete J, González I, Ortega M, Figueroa A, Fritsch R, et al. Tabaquismo y salud mental. Revista médica de Chile. 2003 Aug;131(8):873–80. 14558241

[14] Xu Y. M., Chen H. H., Li F., Deng F., Liu X. B., Yang H. C., et al. (2014). Prevalence and correlates of cigarette smoking among Chinese schizophrenia inpatients receiving antipsychotic mono-therapy. PloS one, 9(2), e88478. doi: 10.1371/journal.pone.0088478 24520390 PMC3919787

[15] Jiang J, See YM, Subramaniam M, Lee J. Investigation of Cigarette Smoking among Male Schizophrenia Patients. PLoS One. 2013;8(8):1–7. doi: 10.1371/journal.pone.0071343 23977021 PMC3744579

[16] Ziaaddini H, Kheradmand A, Vahabi M. Prevalence of cigarette smoking in schizophrenic patients compared to other hospital admitted psychiatric patients. Addict Health. 2009 Summer;1(1):38–42. 24494081 PMC3905500

[17] Krishnadas R, Jauhar S, Telfer S, Shivashankar S, McCreadie RG. Nicotine dependence and illness severity in schizophrenia. Br J Psychiatry. 2012 Oct;201(4):306–12. doi: 10.1192/bjp.bp.111.107953 22878134

[18] Tatar ZB. The effect of the severity of nicotine dependence on psychopathology, insight and illness severity in schizophrenia. Annals of Medical Research. 2018;25(3):349–54.

[19] Boşgelmez Ş, Yıldız M. Şizofreni, Şizoaffektif Bozukluk ve Sanrılı Bozuklukta Sigara Tüketimi ve İlişkili Etmenler. Dusunen Adam. 2017 Sep 1;30(3):233–42.

[20] Molla Z, Dube L, Krahl W, Soboka M. Tobacco dependence among people with mental illness: a facility-based cross sectional study from Southwest Ethiopia. BMC research notes. 2017 Dec;10(1):1–7. doi: 10.1186/s13104-016-2345-3 28716128 PMC5512937

[21] Heatherton TF, Kozlowski LT, Frecker RC, Fagerstrom KO. The Fagerström test for nicotine dependence: a revision of the Fagerstrom Tolerance Questionnaire. British journal of addiction. 1991 Sep;86(9):1119–27. doi: 10.1111/j.1360-0443.1991.tb01879.x 1932883

[22] Buckley TC, Mozley SL, Holohan DR, Walsh K, Beckham JC, Kassel JD. A psychometric evaluation of the Fagerström Test for Nicotine Dependence in PTSD smokers. Addictive behaviors. 2005 Jun 1;30(5):1029–33. doi: 10.1016/j.addbeh.2004.09.005 15893100

[23] Mikami I, Akechi T, Kugaya A, Okuyama T, Nakano T, Okamura H, et al. Screening for nicotine dependence among smoking‐related cancer patients. Japanese journal of cancer research. 1999 Oct;90(10):1071–5. doi: 10.1111/j.1349-7006.1999.tb00679.x 10595734 PMC5926006

[24] Diagnostic Edition F. and statistical manual of mental disorders. Am Psychiatric Assoc. 2013;21.

[25] Manzar MD, Alamri M, Mohammed S, Khan MA, Chattu VK, Pandi-Perumal SR, et al. Psychometric properties of the severity of the dependence scale for Khat (SDS-Khat) in polysubstance users. BMC psychiatry. 2018 Dec;18(1):1–7. doi: 10.1186/s12888-017-1517-6 30340476 PMC6195706

[26] Ayano G, Solomon M, Hibdiye G, Duko B. The epidemiology of tobacco use in Ethiopia: a systematic review and meta-analysis. Journal of Public Health. 2020 Oct 15:1–1.

[27] Latha SP, Kumar MV, Tialam G, Mallepalli PK. Prevalence of nicotine dependence and its impact on quality of life and severity of symptoms in schizophrenic patients. Archives of Mental Health. 2019 Jan 1;20(1):14.

[28] Zhang XY, Liang J, Chen DC, Xiu MH, He J, Cheng W, et al. Cigarette smoking in male patients with chronic schizophrenia in a Chinese population: prevalence and relationship to clinical phenotypes. PloS one. 2012 Feb 7;7(2):e30937. doi: 10.1371/journal.pone.0030937 22347412 PMC3274516

[29] Patkar AA, Gopalakrishnan R, Lundy A, Leone FT, Certa KM, Weinstein SP. Relationship between tobacco smoking and positive and negative symptoms in schizophrenia. The Journal of nervous and mental disease. 2002 Sep 1;190(9):604–10. doi: 10.1097/00005053-200209000-00005 12357094

[30] Meszaros ZS, Dimmock JA, Ploutz‐Snyder RJ, Abdul‐Malak Y, Leontieva L, Canfield K, et al. Predictors of smoking severity in patients with schizophrenia and alcohol use disorders. The American journal on addictions. 2011 Sep;20(5):462–7. doi: 10.1111/j.1521-0391.2011.00150.x 21838846 PMC3156655

[31] Li Y, Hou CL, Ma XR, Zang Y, Jia FJ, Lai KY, et al. Nicotine dependence in community-dwelling Chinese patients with schizophrenia. General psychiatry. 2019;32(1). doi: 10.1136/gpsych-2018-100014 31179421 PMC6551433

[32] Yoshimasu K, Kiyohara C. Genetic influences on smoking behavior and nicotine dependence: a review. Journal of Epidemiology. 2003;13(4):183–92. doi: 10.2188/jea.13.183 12934961 PMC9663414

[33] Rey R, D’amato T, Boyer L, Brunel L, Aouizerate B, Berna F, et al. Nicotine dependence is associated with depression and childhood trauma in smokers with schizophrenia: results from the FACE-SZ dataset. European archives of psychiatry and clinical neuroscience. 2017 Sep;267(6):567–77. doi: 10.1007/s00406-017-0779-9 28389889

[34] An HM, Tan YL, Tan SP, Shi J, Wang ZR, Yang FD, et al. Smoking and serum lipid profiles in schizophrenia. Neuroscience bulletin. 2016 Aug;32(4):383–8. doi: 10.1007/s12264-016-0022-0 27017941 PMC5563778

[35] Mori T, Sasaki T, Iwanami A, Araki T, Mizuno K, Kato T, et al. Smoking habits in Japanese patients with schizophrenia. Psychiatry research. 2003 Sep 30;120(2):207–9. doi: 10.1016/s0165-1781(03)00191-4 14527652

