# Supplementary material for: Prevalence of tobacco dependence and associated factors among patients with schizophrenia attending their treatments at southwest Ethiopia; hospital-based cross-sectional study
Source: PLoS One. 2021 Dec 15;16(12):e0261154. doi: 10.1371/journal.pone.0261154 (PMC8673664; doi:10.1371/journal.pone.0261154)
Supplement: Attachment — Submitted filename: Response letter 2.docx [file pone.0261154.s001.docx]

(1) Thank you for the additional clarification. One of PLOS ONE's publication criteria (https://journals.plos.org/plosone/s/criteria-for-publication#loc-3) states that "Experiments must have been conducted rigorously, with appropriate controls and replication. Sample sizes must be large enough to produce robust results, where applicable. Methods and reagents must be described in sufficient detail for another researcher to reproduce the experiments described."

    Another criteria (https://journals.plos.org/plosone/s/criteria-for-publication#loc-6) states that research must be performed to the highest ethical standards, which includes special precautions for vulnerable groups, such as assessing their capacity to give consent.

    Therefore, we ask that you describe the specific criteria for which the interviewer assessed the participants' capacity to give consent to participate. Unfortunately, stating that it was determined based on the "interviewer's perception" does not provide the level of detail needed for human ethics assurance nor reproducibility. For instance, what was the interviewer's background or expertise that led them to determine capacity to provide consent? What diagnostic criteria were applied? Please provide additional information before we can proceed.

**Author’s Response:** Thank you for your constructive comments. We used nine interviewer's (data collectors) for collecting data from the participants (patients with schizophrenia). The interviewer's background or expertise was that they were all bachelor of degree and masters of degree holders in psychiatry nursing. Hence, we (authors) believe that the interviewer's background or expertise (being psychiatric professional) can led them to determine the capacity of patients to provide consent for the study. The study was done among patients with schizophrenia who were already diagnosed previously as per the Diagnostic Statistical Manual of Mental Disorders 4^th^ and or 5^th^ edition (DSM-IV/DSM-5) and currently attending their treatments at health facility (hospitals). The diagnosis of the patients was first confirmed by reviewing patients’ card prior to starting data collection (interviewing the patients) and also interviewer’s perception for the patient capacity was determined based on the patient level of remission(e.g. for the patient in acute stage of illness or in exacerbation interviewers have excluded them even if they are in partial remission).
