# Supplementary material for: Prevalence of tobacco dependence and associated factors among patients with schizophrenia attending their treatments at southwest Ethiopia; hospital-based cross-sectional study
Source: PLoS One. 2021 Dec 15;16(12):e0261154. doi: 10.1371/journal.pone.0261154 (PMC8673664; doi:10.1371/journal.pone.0261154)
Supplement: Attachment — Submitted filename: Response to Reviwiers.docx [file pone.0261154.s002.docx]

RESPONSE TO EDITOR AND REVIEWER’

1. If the authors have adequately addressed your comments raised in a previous round of review and you feel that this manuscript is now acceptable for publication, you may indicate that here to bypass the “Comments to the Author” section, enter your conflict of interest statement in the “Confidential to Editor” section, and submit your "Accept" recommendation.

Reviewer #1: (No Response)

Reviewer #2: All comments have been addressed

2. Is the manuscript technically sounds, and do the data support the conclusions?
The manuscript must describe a technically sound piece of scientific research with data that supports the conclusions. Experiments must have been conducted rigorously, with appropriate controls, replication, and sample sizes. The conclusions must be drawn appropriately based on the data presented.

Reviewer #1: Partly

Reviewer #2: Partly

**RESPONSE: We accepted the comments and tried to incorporate points that clearly stipulated the findings of the study.**

3. Has the statistical analysis been performed appropriately and rigorously?

Reviewer #1: No

Reviewer #2: I Don't Know

**RESPONSE: In the current study, we used the Statistical Package for Social Science Version 24.0 for data analysis. Descriptive analysis (median, percentage, frequencies, and interquartile range) was used to compute demographic characteristics of participants. In addition, bivariable analysis was used to see the significance of the association. Variables that showed strong association (p-value <0.25) in bivariate analysis were entered into multivariable logistic regressions to identify independently associated variables. Multicollinearity was checked by the variance inflation factor (VIF). Statistical significance was declared at a p-value less than 0.05. The significance of association of the variables was described using AOR with a 95% confidence interval.**

**Unfortunately, there are some editorial errors that occurred during the write up of the result and discussion, such as the report of a female instead of a male in the discussion and exclusion parts. We admitted it and correctly accordingly. Other than this, we did our best and conducted detailed analysis as per the objectives of the study.**

4. Have the authors made all data underlying the findings in their manuscript fully available?
Reviewer #1: Yes

Reviewer #2: Yes

5. Is the manuscript presented in an intelligible fashion and written in Standard English?
PLOS ONE does not copyedit accepted manuscripts, so the language in submitted articles must be clear, correct, and unambiguous. Any typographical or grammatical errors should be corrected at revision, so please note any specific errors here.

Reviewer #1: No

Reviewer #2: No

**RESPONSE: We have accepted and addressed all the English grammatical errors.**

6. Review Comments to the Author
Please use the space provided to explain your answers to the questions above. You may also include additional comments for the author, including concerns about dual publication, research ethics, or publication ethics. (Please upload your review as an attachment if it exceeds 20,000 characters)

Reviewer #1: Prevalence of Tobacco Dependence and Associated Factors among Patients with Schizophrenia Attending Their Treatments at Southwest Ethiopia; Hospital-Based Cross-Sectional Study
Reviewer #1:
1. Abstract In the first sentence the word ‘abused’ and ‘abuser’ is not right. There is no criteria for tobacco abuse in DSM-5.
Authors’ response: Accepted and replaced the word ‘abused’ and ‘abuser’ with the word ‘used’ and ‘user’ respectively.
Re-reviewer response -Even though the word is changed the sentence is not written in a correct English grammar.

**RESPONSE:** **Accepted and corrected as “Tobacco smoking is the most commonly used in patients with mental disorders; patients with schizophrenia are the most frequent users”.**
Result section of the abstract - Concerning the severity of tobacco dependence, 3.5%, 13.8% and 5% of the respondents report high and very high level of tobacco dependence respectively [ this sentence is not correct, misses the word moderate].

**RESPONSE:** **Accepted and edited accordingly as “concerning the severity of tobacco dependence, 3.5%, 13.8% and 5% of the respondents report moderate, high, and very high level of tobacco dependence respectively”.**
2. The introduction part can be shortened. There are a lot of research findings from all the over the world. It should be summarized to the most relevant researches and high quality evidence.
Authors’ response: we accepted the comments and we have shortened the introduction.
Re-reviewer response – accepted
3. Materials and methods please include a statement on the type of the study design.
Authors’ response: Study design was included in the manuscript
Re-reviewers response – accepted
4. Please include some statements about the interviewer’s level of expertise.
Authors’ response: we have included the statements about the interviewer’s level of expertise under ‘data collection procedures and tools’ subtitle of methods section.
Re-reviewers response – accepted
5. And the instrument that has been used to assess tobacco dependence (Fagerstrom Test for Nicotine Dependence (FTND)), was the instrument used in it’s original language or was it translated?
Authors’ response: we used the translated instrument
Re-reviewers response – accepted
6. If it was translated, please specify the process of translation of this or other instruments.
Authors’ response: the process of translation was described under ‘data quality control’ subtitle of methods section
Re-reviewers response – accepted
7. Please put the reference paper which has validated the SDS instrument in Ethiopia.
Authors’ response: Can be added, because it was validated at Mizan, Ethiopia.
Re-reviewers response – if the validation paper was not published in peer reviewed journal, you cannot say that it was validated.
**RESPONSE:** **The instrument was validated in Mizan, the Southwestern part of Ethiopia. This is the references and we have included also in “Manzar MD, Alamri M, Mohammed S, Khan MAY, Chattu VK, Pandi-Perumal SR, et al. Psychometric properties of the severity of the dependence scale for Khat (SDS-Khat) in polysubstance users. BMC Psychiatry. 2018;18(1):1–8.”**

8. Result The 1st paragraph second sentence includes the ‘mean age’.
Authors’ response: we have included
Re-reviewers response – accepted
9. On the next sentences there are repetitions, please correct it.
Authors’ response: we have omitted the repeated sentences
Re-reviewers response – accepted
10. The last sentence the median (IQR) monthly income the IQR is not specified in a range of numbers.
Authors’ response: we accept the comment and corrected accordingly ‘‘the median monthly income of the respondents were 700ETB, which ranges from 100-5000ETB and the interquartile range is 1000.’’
Re-reviewers response – this is still not correct. IQR should be in a range
**RESPONSE:** **IQR was thought to be midspread, the middle 50% of the measure of statistical dispersion, being equal to the difference between 75th and 25th percentiles, or between upper and lower quartiles, and we reported accordingly. Considering the comments we have reported in range.**
11. On the bivariate logistic regression,-- it is univariable logistic regression. There is inconsistency of the result from the table 3 and the above paragraph presented about the significant result on univariable and multivariable logistic regression.
Authors’ response: In our analysis part, for univariable analysis we have used a P –value of 0.25 or less as inclusion criteria for the final model so as not to miss important clinical variables. Accordingly male gender, unemployment, being on treatment for 5years, having a history of admission and frequent admission, presence of physical illness, family history of mental illness and being educated above secondary school. However, for the gender, we mistakenly stated and admit to edit to male.
Re-reviewers response – on the paragraph it is still not edited. Table 4 is not written in a consistent manner. For example the ref. value is sometime on the first row and sometimes on the second row. The analysis you have done is not correlation therefore you should not use the words like “correlations”.

**RESPOSE:** **Accepted ad edited accordingly**
12. Discussion It needs further explanation on recommendation and the uniqueness of this study. Authors’ response: we accept and addressed it
Re-reviewers response – not addressed.

**RESPONSE:** **we have included the statements supporting of the first of the discussion and conclusion.**
On the 4th paragraph 3rd line it says “The notion was indicating the role of genetic factors and nicotine dependence in the pathogenesis of schizophrenia” this needs further explanation. Are you saying that nicotine dependence is causing schizophrenia?

**RESPONSE:** **We accepted the comment and corrected the sentence as** “**the notion was indicating the role of genetic factors in the etiology of smoking behaviour and the high comorbidity between nicotine dependence and schizophrenia” and we are not saying that nicotine dependence is causing schizophrenia. That was an editorial error.**
On the conclusion part it says female gender is associated with tobacco dependence and it should male gender.

**RESPONSE:** **we accepted and edited accordingly. That was an editorial error.**
In general the whole text needs English language revision. The whole manuscript is written in mundane fashion it should be written in attractive way to the readers.

**RESPONSE: we have accepted and addressed all the English language grammar problems.**

Reviewer #2: The authors have addressed all comments, but there are still some issues in the text that must be improved
**RESPONSE:** **We have accepted and addressed all the issues in the text.**
Abstract, Background: „Tobacco smoking is that the most typically employed in patients with mental disorders; among them, patients with schizophrenia area unit the very best users...“ This text is not clear, please, change

**RESPONSE:** **we accepted and changed as “Tobacco smoking is the most commonly used in patients with mental disorders; patients with schizophrenia are the most frequent users”.**
Abstract, results: Concerning the severity of tobacco dependence, 3.5%, 13.8% and 5% of the respondents report high and very high level of tobacco dependence respectively-all three percentages need description., i.e., to which two of the three mentioned percentages do belong high and very high level of ND?

**RESPONSE:** **Accepted and edited accordingly above as “concerning the severity of tobacco dependence, 3.5%, 13.8% and 5% of the respondents report moderate, high, and very high level of tobacco dependence respectively”.**
Results
The mean age (±SD) of the study participants were 33.7(±7.9) year age..-please, delete „age“ at the end of the sentence

**RESPONSE: Accepted and deleted the word “age” from the end of the sentence.**
Table 3. Please, explain what is „frequency “ and percentage“. Does the „frequency“ actually refer to absolute numbers?

**RESPONSE:** **Frequency refers to absolute numbers, while percentage refers to the relative frequency value divided by 100.**
While male gender was associated with tobacco dependence in univariable logistic regression, female gender was associated with tobacco dependence in the final regression analysis. It is difficult to understand, so, please, explain this finding, and mention this also in the discussion.
**RESPONSE: we accepted and edited accordingly. That was an editorial error.**
Discussion
„The results were also higher compared to the finding of the study from Nigeria (20.4)“-percent?

**RESPOSE:** **Accepted and corrected as 20.4%. That was an editorial error.**
References
References are not complete. Please, check and correct carefully all references.
For example, reference No 10 is lacking five additional authors, and the journal name. References 11 and 12 look very much alike, while the No 12 has no journal name

**RESPOSE: 10 corrected,**

7. PLOS authors have the option to publish the peer review history of their article ([what does this mean?](https://journals.plos.org/plosone/s/editorial-and-peer-review-process#loc-peer-review-history)). If published, this will include your full peer review and any attached files.
If you choose “no”, your identity will remain anonymous but your review may still be made public.
**Do you want your identity to be public for this peer review?** For information about this choice, including consent withdrawal, please see our [Privacy Policy](https://www.plos.org/privacy-policy).

Reviewer #1: No

Reviewer #2: No(1)
